# Lack of antidepressant effects of burst-suppressing isoflurane anesthesia in adult male Wistar outbred rats subjected to chronic mild stress

**DOI:** 10.1371/journal.pone.0235046

**Published:** 2020-06-24

**Authors:** Wiebke Theilmann, Marko Rosenholm, Philip Hampel, Wolfgang Löscher, Tomi Rantamäki

**Affiliations:** 1 Department of Pharmacology, Toxicology and Pharmacy, University of Veterinary Medicine Hannover, Hannover, Germany; 2 Division of Pharmacology and Pharmacotherapy, Faculty of Pharmacy, Laboratory of Neurotherapeutics, Drug Research Program, University of Helsinki, Helsinki, Finland; 3 SleepWell Research Program, Faculty of Medicine, University of Helsinki, Helsinki, Finland; Chiba Daigaku, JAPAN

## Abstract

Post-ictal emergence of slow wave EEG (electroencephalogram) activity and burst-suppression has been associated with the therapeutic effects of the electroconvulsive therapy (ECT), indicating that mere “cerebral silence” may elicit antidepressant actions. Indeed, brief exposures to burst-suppressing anesthesia has been reported to elicit antidepressant effects in a subset of patients, and produce behavioral and molecular alterations, such as increased expression of brain-derived neurotrophic factor (BDNF), connected with antidepressant responses in rodents. Here, we have further tested the cerebral silence hypothesis by determining whether repeated exposures to isoflurane anesthesia reduce depressive-like symptoms or influence BDNF expression in male Wistar outbred rats (Crl:WI(Han)) subjected to chronic mild stress (CMS), a model which is responsive to repeated electroconvulsive shocks (ECS, a model of ECT). Stress-susceptible, stress-resilient, and unstressed rats were exposed to 5 doses of isoflurane over a 15-day time period, with administrations occurring every third day. Isoflurane dosing is known to reliably produce rapid EEG burst-suppression (4% induction, 2% maintenance; 15 min). Antidepressant and anxiolytic effects of isoflurane were assessed after the first, third, and fifth drug exposure by measuring sucrose consumption, as well as performance on the open field and the elevated plus maze tasks. Tissue samples from the medial prefrontal cortex and hippocampus were collected, and levels of BDNF (brain-derived neurotrophic factor) protein were assessed. We find that isoflurane anesthesia had no impact on the behavior of stress-resilient or anhedonic rats in selected tests; findings which were consistent—perhaps inherently related—with unchanged levels of BDNF.

## Introduction

Major depression is a highly disabling medical condition that largely contributes to the global disease burden [1]. Presently, it is the most significant risk factor for suicides. Roughly one third of patients with major depression do not respond to prescription antidepressants, but for those who do, the therapeutic effects are evident with a delay of weeks or months of medication. Electroconvulsive therapy (ECT) remains among the most potent treatments for pharmacoresistant depression. Reported response rates to ECT are high, especially for melancholic depression [2–4]. Although relatively safe, ECT may produce adverse effects, such as retrograde amnesia, headache, and nausea [2].

The neurobiological basis of the antidepressant effects of ECT is poorly understood. However, the induction of intrinsic neurotrophic mechanisms, such as activation of BDNF (brain-derived neurotrophic factor) signaling, has been proposed to play a significant role [5–7]. Increase in cortical and hippocampal BDNF mRNA [8–10] and protein [11–13] have been consistently reported after electroconvulsive shock (ECS, an animal model of ECT) treatments. BDNF modulates formation and plasticity of neuronal networks [14–16], and infusions of BDNF into the prefrontal cortex and hippocampus have been shown to mimic the behavioral effects of antidepressants in rodents [17,18]. BDNF has also been implicated in other antidepressant treatments, since the BDNF receptor TrkB (tropomyosin related kinase B) is activated by a variety of pharmacologically diverse antidepressant drugs [19–21], with animals having decreased BDNF-TrkB signaling showing reduced responses to antidepressant treatments [19,22–25].

Rather than mere seizure manifestation or its desired duration, certain post-ictal (i.e. after seizure) events, such as slow wave EEG activity and EEG burst-suppression, have been suggested to predict the efficacy and onset-of-action of ECT [26–30]. General anesthetics, such as isoflurane, dose-dependently produce slowing of EEG activity. When anesthesia deepens, a burst-suppressing EEG pattern is achieved, characterized by bursts of neural activity interrupted by transient periods of electrocerebral silence [31–35]. This similarity to the post-ictal effects of ECT and deep anesthesia prompted research on the exciting possibility that burst-suppressing anesthesia (referred to as “narcotherapy”) would be sufficient to recapitulate the therapeutic effects of ECT in depressed patients. In preliminary clinical studies, isoflurane showed an antidepressant effect comparable to ECT, even after a single dose [31,32,36]. However, subsequent findings remained however inconsistent and did not unequivocally support therapeutic effects of anesthesia in depressed patients [37–40].

Recent clinical and preclinical observations have renewed the interest to investigate the antidepressant effects of deep anesthesia [41]. Weeks et al. demonstrated that a series of ten burst-suppressing isoflurane anesthesia sessions for 15 minutes was comparable to ECT in antidepressant efficacy in patients with medication-refractory depression, and more tolerable than ECT regarding neurocognitive side effects [42]. The same group subsequently reported similar findings with repeated propofol anesthesia [43]. Moreover, we and others have shown that a single isoflurane anesthesia exposure produces antidepressant-like effects in the learned helplessness depression model and in the forced swim test [44,45], while halothane, another anesthetic agent that produces negligible burst-suppression, lacks such effects [45]. Furthermore, isoflurane activates TrkB receptors in a dose-dependent manner, with the most prominent effects observed when burst-suppression is achieved [35,44]. However, activation of TrkB becomes evident even during slight sedation with agents not shown to possess antidepressant effects, indicating that TrkB activation is not *per se* sufficient for antidepressant effects [46], and other mechanisms are likely involved.

To further test “the cerebral silence hypothesis” of ECT and the antidepressant effects of isoflurane anesthesia in particular, we investigated whether repeated isoflurane exposures increase BDNF protein, while ameliorating depressive-like symptoms in Wistar outbred rats (Crl:WI(Han)) subjected to chronic mild stress (CMS). We have recently shown that the depressive-like phenotype in these rats is restored by repeated ECS treatments, which also readily increases BDNF synthesis, while the selective serotonin reuptake inhibitor (SSRI) citalopram was ineffective [47].

## Material and methods

### Animals

A total of 44 adult male Wistar outbred rats (Crl:WI(Han)) were used for the studies (Charles River, Sulzfeld, Germany). Age upon arrival was 9 weeks. Rats were single-housed in Makrolon type III cages on Altromin soft wood granulate. Standard laboratory chow (Altromin 1324 standard diet; Altromin, Lage, Germany) and tap water were provided *ad libitum*, except when CMS procedure required food and/or water deprivation. The controlled 12 h light/12 h dark schedule was only disturbed during stress procedure. All rats were adapted to the laboratory and habituated to handling for at least one week before starting the experiments. Experiments were done in compliance with the European Communities Council Directive of 24 November 1986 (86/609/EEC), and were approved by the animal subjects review board of University of Veterinary Medicine Hannover (LAVES–Lower Saxony State Office for Consumer Protection and Food safety, approval number 12/0871). All efforts were made to minimize pain or discomfort of the animals used. Animals were handled daily, and their general well-being, indicated by grooming behavior and body posture was monitored. Body weight of the animals was measured at least every other day. Two animals that showed more than 20% weight loss over a period of 3 days (humane end point) were excluded from the experiments.

### Chronic mild stress (CMS)

**Fig 1:**
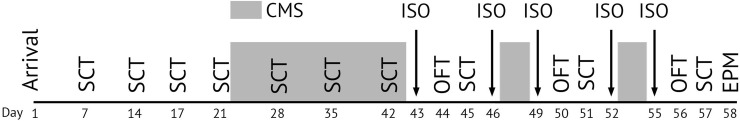
Study timeline showing stress induction, isoflurane anesthesia administration and behavioral testing. CMS = chronic mild stress, EPM = elevated plus maze, ISO = isoflurane anesthesia, OFT = Open field test, SCT = sucrose consumption test.

Rats were exposed to mild stressors at varying time points during light and dark period as described [47,48]. The stressors were delivered daily except when isoflurane/sham treatments were given, and the behavioral performances of the animals were assessed as shown in **Fig 1**. Stressors included periods of (1) continuous light (24 h/d), (2) food deprivation (24 h), (3) water deprivation (14 h), (5) swim sessions in 40°C water (10 minutes in a transparent plexiglas cylinder (50 cm deep, 25 cm diameter) containing 20 cm of water), (6) swim sessions in 15°C water (5 minutes in similar conditions to swim sessions in 40°C water), (7) wet bedding (16 h, 300 ml of tap water on Altromin soft wood granulate), (8) restraint stress (30 min) and (9) social crowding (four rats in one Makrolon type III cage). No stress was applied on the days of behavioral testing or isoflurane/sham administrations. Control rats were left undisturbed and handled regularly.

### Weight measurement

Body weight was monitored during the course of experiments as a measure for general health. A reduction in body weight or a diminished weight gain reflects a reduced well-being of the rats [49].

### Sucrose consumption test (SCT)

Hedonic deficits induced by CMS can be measured as a decrease in consumption or preference for sweet solution [50]. During test sessions rats had free access to a bottle of 1% sucrose solution and a bottle of tap water for 14 h. No food or water deprivation was performed before testing. Animals were habituated to the testing in three habituation trials. The position of the bottles was switched after every test session to avoid possible effects of side preference in drinking behavior. The individualized acquisition of sucrose intake provides the opportunity to select between anhedonic-like rats (stress responders) and hedonic-like rats (stress non-responders). Anhedonic- or hedonic-like behavior is based on the individual amount of sucrose solution intake. Rats showing >25% within-subject decrease in sucrose consumption were considered anhedonic while rats showing <10% within-subject decrease in sucrose consumption were considered hedonic [1]. Animals not belonging to either criterion were considered as unclassifiable. Sucrose consumption was measured 3 to 5 times before CMS. After isoflurane anesthesia, alterations in anhedonic-like behavior were assessed by estimating within subject changes in sucrose consumption. According to Christensen et al., positive treatment responders were anhedonic-like animals showing >20% within-subject increase in sucrose consumption, whereas non-responders show <20% within-subject increase in sucrose consumption. The mean of the sucrose consumption determined in these trials was set as 100% and considered baseline level.

### Open field test

The open field test is a routine method to measure locomotor activity and anxiety-like behavior in rodents [51]. The test was performed in a round arena made of black PVC (diameter 80 cm) which was divided into three zones (center, inner, outer). The animals were placed individually in the center of the open field. Distance moved and time spent in the center of the open field was recorded for 5 min and analyzed with EthoVision®XT7 software (Noldus Information Technology, Wagening, Netherlands).

### Elevated plus maze test

The elevated-plus maze measures the level of anxiety in rodents [52]. The apparatus was constructed with black plastic. It comprises two open arms (50x10 cm), two enclosed arms (50x10x30 cm), and a central platform (10x10 cm). The configuration has the shape of a plus sign, and the apparatus is elevated 80 cm above the floor level. At the beginning of the test, rats were placed on the central platform always facing the same closed arm. The behavior of rats in the test was analyzed for 5 min using the EthoVision®XT7 software (Noldus Information Technology, Wagening, Netherlands). Time spent in different sections of the maze (open and closed arms) and the frequency of entries into open and closed arms were assessed.

### Isoflurane exposure

Rats were randomly allocated to the treatment groups: control + sham (N = 8), control + isoflurane (N = 8), CMS + sham (N = 14), CMS + isoflurane (N = 14). Rats were placed into an anesthesia box and exposed to isoflurane (induction: 4% for 2 min; maintenance: 2% for 13 min; airflow of 1.0 l/min). This isoflurane dosing regimen produces a rapid burst-suppression EEG state highly reliably in both rodents and humans [31,34,35,45,53]. Sham animals were kept in the anesthesia boxes for 2 min without isoflurane. To measure the behavioral outcomes in SCT and open field tests in between administrations, and to model the preliminary clinical studies demonstrating isoflurane’s antidepressant effect [32,42], a single treatment was given once every third day over 15 days (= 5 total treatments).

### BDNF ELISA

After the behavioral experiments, the animals were euthanized by decapitation after a brief exposure to carbon dioxide. Tissue samples from the medial prefrontal cortex and hippocampus were rapidly dissected and snap-freezed. BDNF protein levels were analyzed using a commercial BDNF ELISA kit (Quantikine^®^ ELISA Kit, catalog #DBD00, R&D Systems Europe Ltd., Abingdon, UK). The samples were homogenized in NP++ lysis buffer (137 mM NaCl, 20 mM Tris, 1% NP-40, 10% glycerol, Pierce™ Protease and Phosphatase Inhibitor tablets (Thermo Fisher Scientific, Waltham, MA), 48 mM NaF), incubated on ice for 15 minutes, centrifuged (16,000 g, 15 min, 4°C), and the supernatants were collected for further processing. The samples were acidified to pH 3 with 1 M HCl, followed by neutralization with 1 M NaOH. The samples were loaded on a pre-coated (with monoclonal BDNF antibody) and pre-blocked 96-well plate containing serial diluted BDNF standards and hippocampal samples from adult male conditional Bdnf^-/-^ knockout [54,55] and wild-type mice (kindly provided by Dr. Maribel Rios), and incubated for 2 hours at RT. The plate was then incubated with HRP-conjugated secondary monoclonal BDNF antibody for 1 hour at RT, followed by three washes with provided wash buffer, and then incubated with color reagents (hydrogen peroxide and chromogen). The reaction was stopped with 2 M H_2_SO_4_ after a 30-minute incubation, and the plate was read for absorbance in 450 nm. The obtained results were normalized to total protein concentrations of each sample.

### Statistics

Data are shown as mean ± SEM (standard error of mean). Two-way analysis of variance (ANOVA) (two categorical independent variables), repeated measures ANOVA followed by Sidak’s multiple comparisons test, or Student’s unpaired t-test were used for statistical evaluation (Prism 7 software, GraphPad (La Jolla, CA, USA). A *P*<0.05 was considered statistically significant. Details of statistical tests are shown in S1 Table.

## Results

### Chronic mild stress induced alterations in sucrose consumption and body weight in rats

Chronic mild stress (CMS) is considered one of the most valid animal models of depression [56]. In this model, as the name implies, the animals are repeatedly subjected to various stressors during a course of several weeks, which may induce depression-like phenotypes, most notably anhedonia (e.g. reduced consumption of sweetened solution). The strength of this model is that animals respond to chronic, but not acute, administration of antidepressant drugs (and to ECS), as compared to drugs without clinical antidepressant properties that show no effects [56]. We recently employed this model using a stress-sensitive substrain of male outbred Wistar rats (Crl:WI(Han)) [47]. Repeated ECS ameliorated depression-like phenotypes induced by CMS and significantly increased BDNF levels. Additionally, the SSRI citalopram had negligible effects on both phenotype and BDNF levels [47].

**Fig 2:**
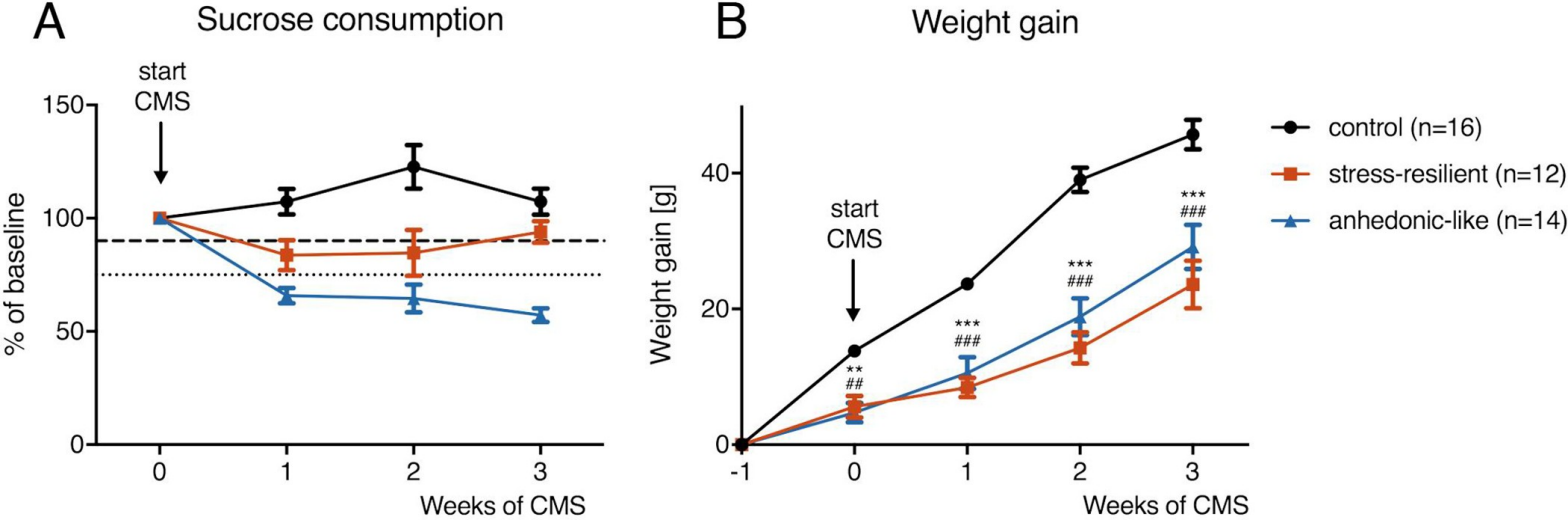
Effects of chronic mild stress on sucrose consumption and body weight. **A**). Sucrose consumption survey over 3 weeks of CMS demonstrates that 53.8% (14/26) of the animals responded to stress with anhedonic behavior characterized by a >25% within-subject decrease in sucrose consumption (small dashed lines). 46.2% (12/26) of the CMS exposed animals were classified stress-resilient, showing a within-subject decrease in sucrose intake of <10% (wide dashed lines). Hedonic-like behavior was present in all unstressed control rats. **B**) Stressed rats gained significantly less body weight during three weeks of CMS compared to controls (Repeated measures ANOVA: F_2,39_ = 26.44, P<0.0001). CMS = chronic mild stress. Data is shown as mean ± SEM. **<0.01, ***<0.001 (control vs. anhedonic-like), ##<0.01, ###<0.001 (control vs. stress-resilient), repeated measures ANOVA followed by Sidak’s multiple comparisons test.

As shown recently in this rat strain (Neyazi et al. 2018), during three weeks of CMS rats begin to segregate based on behavioral change to anhedonic-like or hedonic-like behavioral groups. According to Christensen et al. (2011), anhedonic-like animals are expected to show a > 25% within-subject decrease in sucrose intake, whereas hedonic-like rats are expected to show a < 10% within-subject reduction in sucrose intake. Animals not responding to either criterion are considered unclassifiable. In the present experiments, anhedonic-like behavior was present in 53.8% (14/26) of the animals, whereas hedonic-like behavior was detected in the remaining animals (12/26) (**Fig 2A**). None of the unstressed control animals showed anhedonic-like behavior. The average weight gain of the rats during three weeks of CMS was significantly lower compared to weight gain of unstressed controls (Repeated measures ANOVA: F_2,39_ = 26.44, P<0.0001) (**Fig 2B**).

### Lack of effects of repeated isoflurane anesthesia on behavioral changes induced by CMS

**Fig 3:**
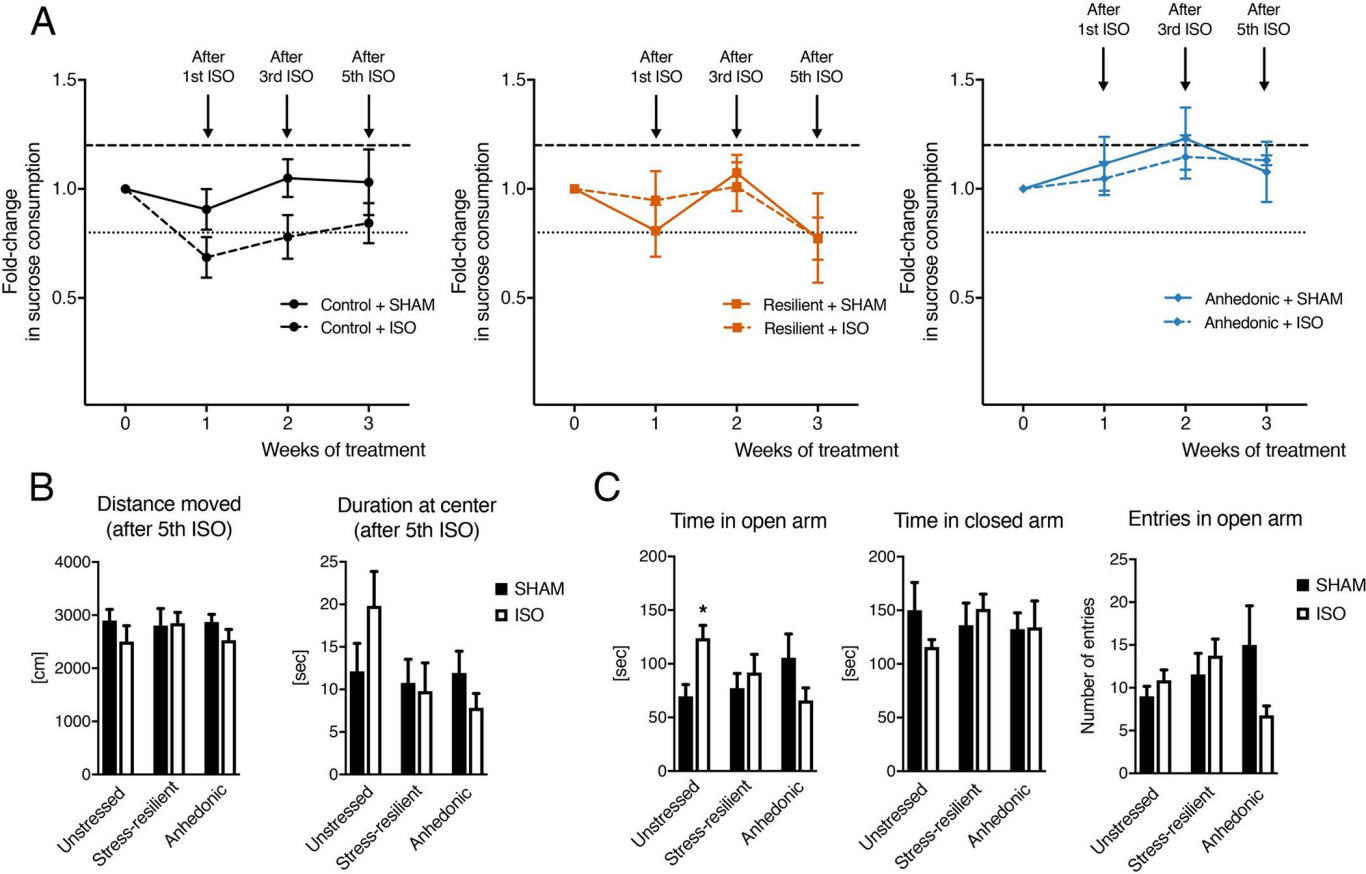
Lack of antidepressant effects of isoflurane anesthesia in a chronic mild stress model of depression. **A)** Changes in sucrose consumption of control, stress-resilient, and anhedonic animals 2 days after isoflurane administrations following CMS exposure. **B)** Distance traveled and time spent at arena center in open field test 24 hours after 5th isoflurane administration. **C)** Results in elevated plus maze test 3 days after 5th isoflurane exposure. ISO = isoflurane anesthesia. Data is shown as mean ± SEM. *<0.05, two-way ANOVA.

The animals were next subjected to 15-minute burst-suppressing isoflurane anesthesia (induction: 4%; maintenance: 2%) [34,35] or sham anesthesia every third day over a 15-day period for a total of 5 consecutive treatments (**Fig 1**). Antidepressant and anxiolytic effects of isoflurane were assessed after the first (rapid), third, and fifth drug exposure using the sucrose consumption, the open field, and the elevated plus maze tests. Based on the criteria by Christensen et al., positive treatment responders were considered as anhedonic-like animals showing >20% within-subject increase in sucrose intake, whereas non-responders were considered to show <20% within-subject increase in sucrose intake [1]. Exposure to isoflurane exerted no significant effects on sucrose consumption in anhedonic-like (Repeated measures ANOVA: F_1,12_ = 0.06915, P = 0.80), stress-resilient (F_1,10_ = 0.04871, P = 0.83) or sham rats (F_1,14_ = 4.117, P = 0.06) (**Fig 3A**). If anything, the sucrose consumption observed in non-stressed rats was reduced by isoflurane treatment, although this effect was not significant. Sucrose consumption in anhedonic-like groups remained low throughout the experiments, indicating that a depressive-like phenotype induced by the CMS protocol was sustained throughout the experiments (**S1 Fig**). In addition, isoflurane produced only minor behavioral effects in the open field or the elevated plus maze tests (**Fig 3B and 3C, S2 Fig**). Isoflurane exerted contrasting effects in control and anhedonic-like groups on number of entries (Two-way ANOVA, treatment x phenotype: F_2,35_ = 3.579, P = 0.0385) and time spent in open arm (F_2,35_ = 5.845, P = 0.0065) of the elevated plus-maze (**Fig 3C**), and decreased overall locomotor activity in the open field task after the first (Two-way ANOVA, treatment effect: F_1,36_ = 5.693, P = 0.0224) and third (F_1,36_ = 4.212, P = 0.0475) isoflurane administrations (**S2 Fig**).

### Brain BDNF levels remain unaltered after CMS and isoflurane administrations

**Fig 4:**
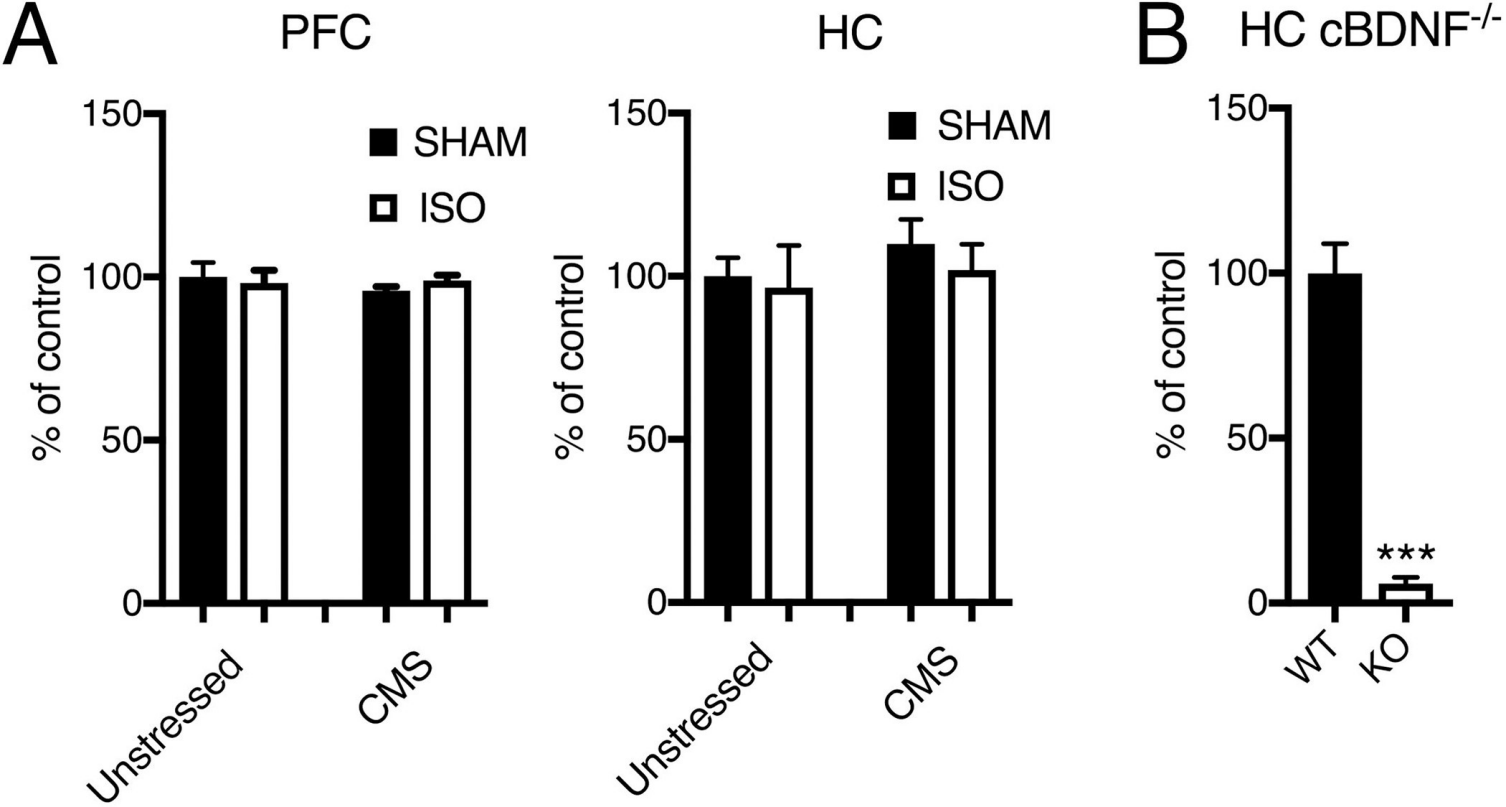
CMS and isoflurane anesthesia have no significant effect on BDNF protein levels in the rat medial prefrontal cortex (PFC) and hippocampus (HC). (B) Assay specificity was determined with cortical samples of conditional BDNF^-/-^ mice that showed negligible signal in comparison to wild-type littermates. CMS = chronic mild stress, ISO = isoflurane anesthesia. Data is shown as mean ± SEM. ***<0.001, Student’s unpaired t-test.

After the behavioral experiments the animals were euthanized and samples collected from the medial prefrontal cortex and hippocampus to determine BDNF protein levels. To test the specificity of the ELISA assay, we also determined BDNF expression in hippocampal homogenates obtained from adult male conditional Bdnf^-/-^ mice and their wild-type littermates. Results from BDNF protein analysis show negligible effects of the animals’ response to CMS (Two-way ANOVA, phenotype effect, PFC: F1,15 = 0.3992, P = 0.54; HC: F1,28 = 0.7168, P = 0.40), and isoflurane anesthesia (Two-way ANOVA, treatment effect, PFC: F1,15 = 0.05878, P = 0.81; HC: F1,28 = 0.4036, P = 0.53 (**Fig 4A**).

## Discussion

Post-ictal EEG suppression has been proposed to predict the antidepressant effects of ECT [26–30]. Like ECT, the volatile anesthetic isoflurane causes EEG burst-suppression in humans and rodents when adequate dosing is applied [32,35]. Clinical and preclinical evidence indicates that such burst-suppressing isoflurane anesthesia ameliorates depressive symptoms in patients, and elicits antidepressant-like effects in rodents [31,32,36,42,44,45,57]. Already a single brief isoflurane anesthesia has demonstrated antidepressant-like effects in the forced swim test and learned helplessness model in rodents [44,45]. Amelioration of anhedonic behavior was also observed after a single isoflurane anesthesia exposure in a mouse model of CMS [57]. Here, we utilized a CMS model in a stress-sensitive substrain of rats that respond to ECS, but not citalopram [47] in order to further test the antidepressant-like effects of repeated burst-suppressing isoflurane anesthesia. The dosing of isoflurane was selected based on our earlier data to achieve reliable burst-suppression pattern and TrkB signaling [35], which is one of the main pathways targeted by antidepressants [58]. A subset of rats responded to stress by showing reduced sucrose consumption (a marker of anhedonia), while some of the animals remained stress-resilient, a finding previously observed [1,47]. Unexpectedly, we found no significant behavioral changes in any of the treatment groups after isoflurane administrations at any point during the course of the experiments.

Repeated exposures to anesthesia had no impact on BDNF levels, a finding that contrasts numerous studies showing that all other antidepressants increase BDNF synthesis [58]. During ECT practice, an electric current is delivered onto the scalp of the patient under anesthesia, which leads to transient epileptiform EEG activity. This robust increase in neuronal activity likely underlies the stimulatory effects of ECT on BDNF levels, since various types of neuronal stimuli–especially generalized convulsions–have been shown to increase BDNF synthesis [14,59–61]. Isoflurane shares the capability to induce electrocerebral silence with ECT, but it often brings no preceding convulsions or seizure activity. Indeed, the effects of anesthetics, such as isoflurane, on brain and blood BDNF levels generally remain negligible or even decrease [44,62–70]. Anesthesia also blocks rTMS (repetitive transcranial magnetic stimulation) induced BDNF synthesis [71].

Despite anesthesia producing a state of widespread depression in the CNS, paradoxical neuronal excitation has been reported with diverse anesthetics, especially when the concentration of anesthetic is low [72,73]. This is particularly well exemplified by ketamine, a rapid-acting antidepressant that is used in subanesthetic dosing to treat depression. Ketamine provokes cortical excitability by increasing glutamatergic neurotransmission [74,75]. This excitatory response has been shown to be required for its antidepressant-like effect in rodents [76,77]. Furthermore, an increase in cortical excitability after ketamine administration has been associated with a positive antidepressant treatment response in patients [78]. More recently, we have shown that subanesthetic doses of nitrous oxide, a putative rapid-acting antidepressant [79], readily up-regulates BDNF synthesis and several other markers of neuronal excitation [46]. Notably, both subanesthetic ketamine and nitrous oxide evoke slow wave activity, as measured by EEG, after the peak of their pharmacological effects, resembling the post-ictal state following ECT. Rapid-acting antidepressants may therefore require both a phase of neuronal excitation, and emergence of slow wave activity to elicit their therapeutic effects [80]. It’s tempting to speculate that isoflurane’s antidepressant effects may be dependent on the treatment protocol´s (unpredictable) capability to induce sufficient neuronal excitation, BDNF synthesis, and EEG silencing. Indeed, isoflurane has been shown to elicit antidepressant-like effects in a mouse model of CMS using an administration protocol that also increased BDNF expression [57]. Additionally, isoflurane is known to produce occasional excitatory responses and behavioral hyperactivity/agitation particularly during anesthesia induction and emergence [81–83]. Excitation may also occur during deep burst-suppressing anesthesia, where isoflurane increases cortical excitability in response to various stimuli [84–86]. Validation of anesthesia treatment regimens capable of producing these effects should therefore be considered in future studies [41].

## Supporting information

S1 Fig Sucrose consumption of the anhedonic rats remain significantly lower than in control group throughout the experiments. CMS = chronic mild stress. Data is shown as mean ± SEM. ***<0.001, *<0.05, Repeated measures ANOVA followed by Sidak’s multiple comparisons test.
(PDF)

S2 Fig Effects of isoflurane in open field test 24 hours after 1st and 3rd isoflurane administration. ISO = isoflurane anesthesia. Data is shown as mean ± SEM.
(PDF)

S1 Table Statistical analyses and *n* numbers. (PDF)
