# Supplementary material for: Lack of antidepressant effects of burst-suppressing isoflurane anesthesia in adult male Wistar outbred rats subjected to chronic mild stress
Source: PLoS One. 2020 Jun 24;15(6):e0235046. doi: 10.1371/journal.pone.0235046 (PMC7313995; doi:10.1371/journal.pone.0235046)
Supplement: S1 Fig — CMS = chronic mild stress. Data is shown as mean ± SEM. ***<0.001, *<0.05, Repeated measures ANOVA followed by Sidak’s multiple comparisons test. (PDF) [file pone.0235046.s001.pdf]

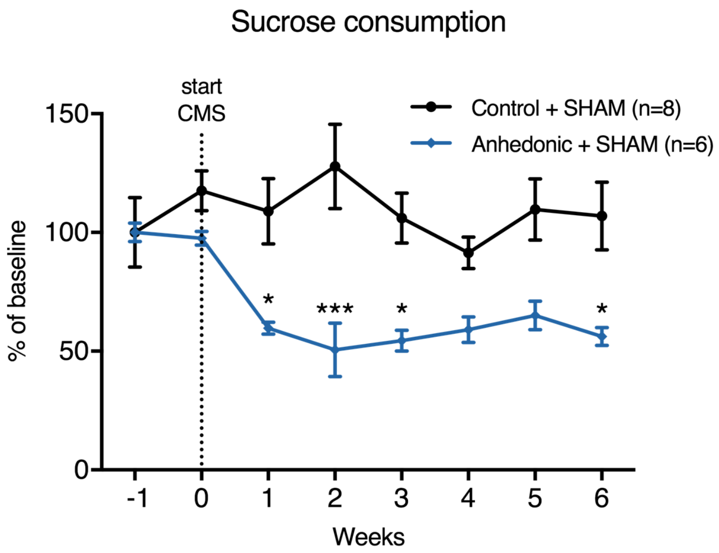

Figure S1. Sucrose consumption of the anhedonic rats remain significantly lower than in control group throughout the experiments. CMS = chronic mild stress. ***<0.001, *<0.05, Repeated measures ANOVA followed by Sidak’s multiple comparisons test.
