# Supplementary material for: Lack of antidepressant effects of burst-suppressing isoflurane anesthesia in adult male Wistar outbred rats subjected to chronic mild stress
Source: PLoS One. 2020 Jun 24;15(6):e0235046. doi: 10.1371/journal.pone.0235046 (PMC7313995; doi:10.1371/journal.pone.0235046)
Supplement: S2 Fig — ISO = isoflurane anesthesia. Data is shown as mean ± SEM. (PDF) [file pone.0235046.s002.pdf]

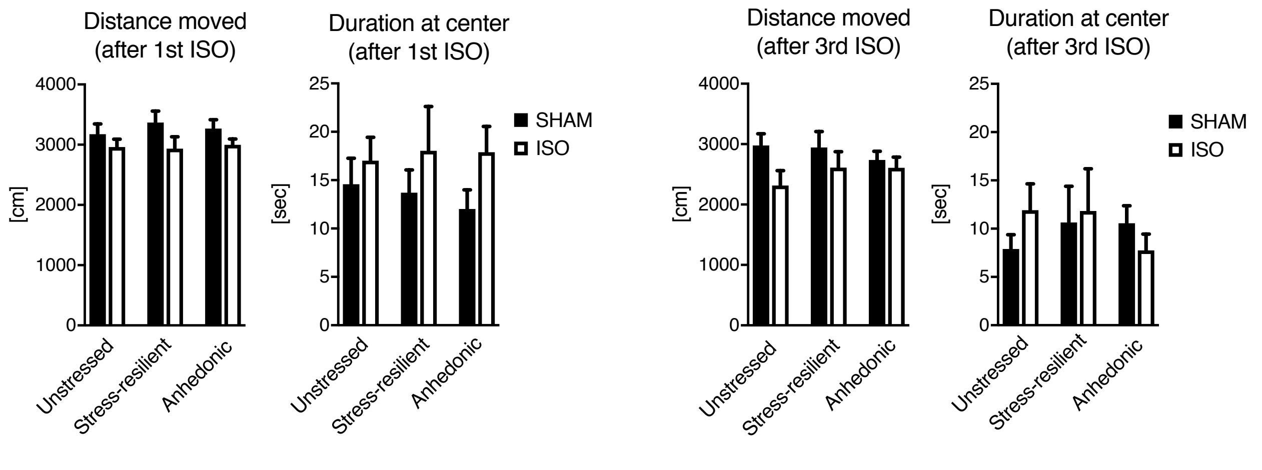

Figure S2. Distance moved and duration spent at arena center in open field test 24 hours after 1st and 3rd isoflurane administration. ISO = isoflurane anesthesia. Data is shown as mean ± SEM.
