# Supplementary material for: Lack of antidepressant effects of burst-suppressing isoflurane anesthesia in adult male Wistar outbred rats subjected to chronic mild stress
Source: PLoS One. 2020 Jun 24;15(6):e0235046. doi: 10.1371/journal.pone.0235046 (PMC7313995; doi:10.1371/journal.pone.0235046)
Supplement: S1 Table — (PDF) [file pone.0235046.s003.pdf]

Figure 2	n	Statistical test	Significance	
**A** Sucrose consumption test				
Control vs. Stress-resilient vs. Anhedonic-like	16, 12, 14	no statistical testing		
**B** Body weight gain				
Control vs. Stress-resilient vs. Anhedonic-like	16, 12, 14	Repeated measures ANOVA	F (2, 39) = 26.44, P<0.0001	***
		Sidak's multiple comparisons test		
		Control vs. Stress-resilient		
		0 (Weeks of CMS)	P=0.0084	**
		1	P<0.0001	***
		2	P<0.0001	***
		3	P<0.0001	***
		Control vs. Anhedonic-like		
		0 (Weeks of CMS)	P=0.0018	**
		1	P<0.0001	***
		2	P<0.0001	***
		3	P<0.0001	***
**Figure 3**	**n**	**Statistical test**	**Significance**	
**A** Sucrose consumption test				
Control (Sham vs. Isoflurane)	8	Repeated measures ANOVA	F (1,14) = 4.117, P=0.06	ns
Stress-reslient (Sham vs. Isoflurane)	7, 5	Repeated measures ANOVA	F (1,10) = 0.04871, P=0.83	ns
Anhedonic-like (Sham vs. Isoflurane)	6, 8	Repeated measures ANOVA	F (1,12) = 0.06915, P=0.80	ns
**B** Open Field (after 5th Isoflurane)	5-8			
Distance moved		Two-way ANOVA		
		Treatment	F (1, 36) = 1.272, P=0.27	ns
		Phenotype	F (2, 36) = 0.1545, P=0.86	ns
		Interaction	F (2, 36) = 0.4243, P=0.66	ns
Duration at center		Two-way ANOVA		
		Treatment	F (1, 36) = 0.1152, P=0.74	ns
		Phenotype	F (2, 36) = 2.553, P=0.09	ns
		Interaction	F (2, 36) = 2.076, P=0.14	ns
**C** Elevated plus-maze (After 5th Isoflurane)	4-8			
Time in open arm		Two-way ANOVA		
		Treatment	F (1, 35) = 0.6392, P=0.43	ns
		Phenotype	F (2, 35) = 0.462, P=0.64	ns
		Interaction	F (2, 35) = 5.845, P=0.0065	**
		Sidak's multiple comparisons test (Sham vs. ISO)		
		Unstressed	P=0.0191	*
		Stress-resilient	P=0.9027	ns
		Anhedonic-like	P=0.1604	ns
Time in closed arm		Two-way ANOVA		
		Treatment	F (1, 35) = 0.1133, P=0.74	ns
		Phenotype	F (2, 35) = 0.1462, P=0.86	ns
		Interaction	F (2, 35) = 0.7676, P=0.47	ns
Entries in open arm		Two-way ANOVA		
		Treatment	F (1, 35) = 0.5711, P=0.45	ns
		Phenotype	F (2, 35) = 0.7013, P=0.50	ns
		Interaction	F (2, 35) = 3.579, P=0.0385	*
		Sidak's multiple comparisons test (Sham vs. ISO)		
		Unstressed	P=0.8881	ns
		Stress-resilient	P=0.9083	ns
		Anhedonic-like	P=0.0348	*
**Figure 4**	**n**	**Statistical test**	**Significance**	
BDNF ELISA				
PFC	3-7	Two-way ANOVA		
		Treatment	F (1, 15) = 0.05878, P=0.81	ns
		Phenotype	F (1, 15) = 0.3992, P=0.54	ns
		Interaction	F (1, 15) = 0.8613, P=0.37	ns
HC	7-10	Two-way ANOVA		
		Treatment	F (1, 28) = 0.4036, P=0.53	ns
		Phenotype	F (1, 28) = 0.7168, P=0.40	ns
		Interaction	F (1, 28) = 0.06675, P=0.80	ns
cBDNF -/-	3	Student's unpaired t-test	P=0.0005	***
**Figure S1**	**n**	**Statistical test**	**Significance**	
Sucrose consumption test				
Control-SHAM vs. Anhedonic-SHAM	8, 6	Repeated measures ANOVA	F (1, 12) = 10.87, P=0.0064	**
		Sidak's multiple comparisons test		
		0 (Week)	P=0.8674	ns
		1	P=0.0258	*
		2	P<0.0001	***
		3	P=0.0161	*
		4	P=0.3351	ns
		5	P=0.0571	ns
		6	P=0.0192	*

Table S1. Statistical analyses and *n* numbers

Figure S2	n	Statistical test	Significance	
**A** Open Field (after 1st Isoflurane)	5-8			
Distance moved		Two-way ANOVA		
		Treatment	F (1, 36) = 5.693, P=0.0224	*
		Phenotype	F (2, 36) = 0.1577, P=0.85	ns
		Interaction	F (2, 36) = 0.2511, P=0.78	ns
Duration at center		Two-way ANOVA		
		Treatment	F (1, 36) = 3.43, P=0.07	ns
		Phenotype	F (2, 36) = 0.06511, P=0.94	ns
		Interaction	F (2, 36) = 0.209, P=0.81	ns
**B** Open Field (after 3rd Isoflurane)	5-8			
Distance moved		Two-way ANOVA		
		Treatment	F (1, 36) = 4.212, P=0.0475	*
		Phenotype	F (2, 36) = 0.1824, P=0.83	ns
		Interaction	F (2, 36) = 0.7872, P=0.46	ns
Duration at center		Two-way ANOVA		
		Treatment	F (1, 36) = 0.1314, P=0.72	ns
		Phenotype	F (2, 36) = 0.284, P=0.75	ns
		Interaction	F (2, 36) = 0.8751, P=0.43	ns

Table S1. Statistical analyses and *n* numbers (*continued*)
